# Integrated Brain Circuits: Neuron-Astrocyte Interaction in Sleep-Related Rhythmogenesis

**DOI:** 10.1100/tsw.2010.130

**Published:** 2010-08-17

**Authors:** Michael M. Halassa, Marco Dal Maschio, Riccardo Beltramo, Philip G. Haydon, Fabio Benfenati, Tommaso Fellin

**Affiliations:** ^1^ Department of Psychiatry, Massachusetts General Hospital, Boston, MA, USA; ^2^ Department of Psychiatry, McLean Hospital, Belmont, MA, USA; ^3^ Department of Brain and Cognitive Science, Massachusetts Institute of Technology, Cambridge, USA; ^4^ Department of Neuroscience and Brain Technologies, Italian Institute of Technology (IIT), Genova, Italy; ^5^ Department of Neuroscience, Tufts University, Boston, MA, USA

**Keywords:** glia, sleep, slow oscillations, adenosine, A1 receptors, cortical rhythms

## Abstract

Although astrocytes are increasingly recognized as important modulators of neuronal excitability and information transfer at the synapse, whether these cells regulate neuronal network activity has only recently started to be investigated. In this article, we highlight the role of astrocytes in the modulation of circuit function with particular focus on sleep-related rhythmogenesis. We discuss recent data showing that these glial cells regulate slow oscillations, a specific thalamocortical activity that characterizes non-REM sleep, and sleep-associated behaviors. Based on these findings, we predict that our understanding of the genesis and tuning of thalamocortical rhythms will necessarily go through an integrated view of brain circuits in which non-neuronal cells can play important neuromodulatory roles.

## INTRODUCTION

One of the most intriguing questions of modern neuroscience is why animals spend much of their lives in the global state of behavioral inactivity we call sleep. From an evolutionary standpoint, sleep appears to be both ancient and pervasive across the animal kingdom[1,2]. Although the functions of sleep are currently poorly understood[3,4,5], a growing body of literature suggests a role for sleep in memory consolidation[6,7,8,9]. In humans, a period of post-training sleep correlates with enhanced declarative and procedural memory retention compared to equal time spent in wakefulness[10], and, under specific experimental conditions, the consolidation of previously memorized cues is enhanced by reintroducing those cues during sleep[11,12]. In nonhuman animals, sleep enhances procedural memories[13] and cortical plasticity[14,15].

A number of hypotheses have been proposed to explain the involvement of sleep in off-line memory consolidation, including *synaptic homeostasis* and *memory reactivation*. Synaptic homeostasis suggests that, overall, sleep scales down synaptic weights such that weak memory traces are eliminated, thus enhancing the signal-to-noise ratio of information encoded in the brain[16,17]. Memory reactivation, on the other hand, suggests that during sleep, awake neuronal ensemble activity is replayed, leading to synaptic potentiation in circuits relevant to newly acquired memory traces[18,19]. Both of these hypotheses have strong experimental support[16,18,20,21,22,23,24], and it may be that both processes occur during sleep to ensure that relevant memory traces are boosted, while weak memory traces are eliminated.

Although recent experiments have shown a role for coherence between the amygdala and prefrontal cortex during rapid eye movement (REM) sleep in fear memory consolidation[25], the role of slow wave sleep (SWS) in memory consolidation has been more intensely studied (for review, see [6]). Network activity during SWS is thought to mediate many of the functions attributed to sleep in memory consolidation[10,26,27,28]. In SWS, the thalamocortical system is dominated by stereotyped field potential oscillations in the 0.5- to 4-Hz frequency range, which includes the slow (<1 Hz) oscillation and delta frequencies (1–4 Hz). Analytically described by Steriade and colleagues 17 years ago[29,30,31], the slow oscillation is now thought to be one of the primary organizers of network activity in the thalamocortical system[32] and possibly the key player in off-line memory consolidation during sleep[10,21,26]. In the remainder of this review, we will first discuss the neuronal mechanisms underlying slow oscillations, highlighting the role of dynamic cortico- and thalamocortical coupling to generate this network activity. Second, we will discuss the concept of “integrated brain circuits”, highlighting the involvement of astrocytes in regulating neuronal excitability and synaptic transmission, and discussing the potential impact these processes could have on brain network activity. Finally, we will present recent evidence demonstrating that the slow oscillation is a rhythm arising from integrated brain circuit activity, where astrocytes exert powerful modulatory effects on neurons to impact the generation of slow oscillations.

## NEURONAL SUBSTRATES OF SLOW OSCILLATIONS

From a phenomenological point of view, the slow oscillation is an electrophysiological process that is observed at multiple levels of organization in the brain during natural sleep and under certain forms of anesthesia[32,33]. In the human electroencephalogram (EEG), stage 2 sleep is characterized by K-complexes representing single slow oscillation cycles[34,35]. As sleep deepens, K-complexes become more frequent and, ultimately, slow oscillations occupy a greater proportion of the EEG, which is characteristic of SWS. On a large spatial scale, these oscillations have been described as traveling waves originating at a higher probability from prefrontal regions in the sleeping human brain[36]. At smaller scales, slow oscillation activity has complex spatial dynamics[37,38]. Slow oscillations are highly synchronized in the ipsi- and controlateral hemispheres, most likely through the activation of callosal projections[39] and are not limited to neocortical regions, but are also observed in the paleocortex[40], the hippocampus[41,42], and the thalamus[43,44,45,46].

Anesthetized preparations have greatly enhanced our understanding of the neuronal underpinnings of the slow oscillation. Pioneering work by Steriade and colleagues has shown that identified neurons in a number of different cortical areas fluctuate between depolarized (UP) and hyperpolarized (DOWN) states[29,30,31]. The UP state is characterized by barrages of synaptic activity, a plateau depolarization, and action potential firing, while the DOWN state is characterized by cell hyperpolarization and silence[30] (Fig. 1). These membrane potential fluctuations are tightly synchronized to the local field potential, in which waves of field potential slow oscillations trigger precise sequential firing of pyramidal neurons, in particular those in layer V[38], suggesting the presence of local cortical activity motifs. The propagation of slow oscillations, at least in the auditory cortex, seems to initiate in layer V[47,48] (but see [49]) and then propagates to the more superficial layers in contrast to the spread of sensory-evoked activity, which initiates in layer IV[48].

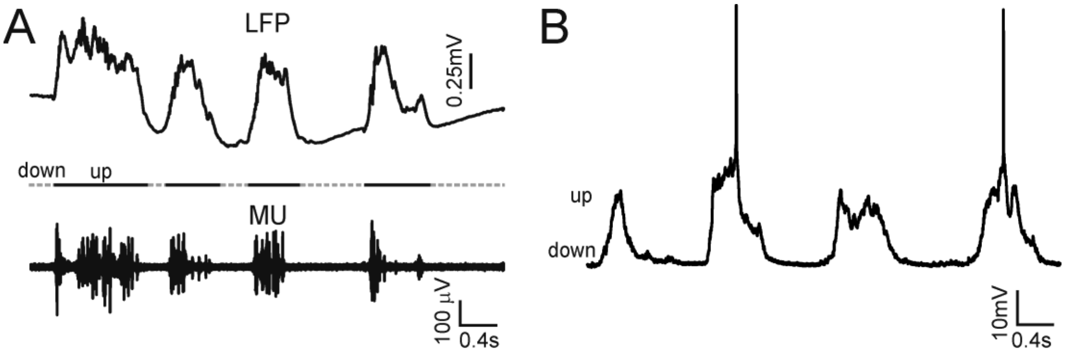

**FIGURE 1.** UP- and DOWN-state transitions during slow oscillation activity. (A) Extracellular local field potential (LFP, top) and multiunit (MU, bottom) recordings showing slow oscillations in the neocortex of urethane-anesthetized mice. Note the recurrent transitions from the UP to the DOWN state at low frequency (<1 Hz). Action potential firing (see MU trace) occurs synchronously among different cortical neurons only during the UP state. (B) Patch-clamp, current-clamp recording from a cortical neuron *in vivo* showing membrane potential changes during slow oscillations.

As mentioned previously, not only cortical, but also thalamocortical cells and neurons of the thalamic reticular nucleus (TRN) exhibit slow oscillations at the cellular level[43,44,45,46]. Interestingly, although the isolated cortical slices and cortical slabs can generate slow oscillations independently of the thalamus[30,47,50,51], there has been no formal quantitative analysis of the characteristics of cortical slow oscillations in the absence of the thalamus[52]. A recently proposed view of the slow oscillation suggests that this rhythm is generated by a synaptic-based cortical oscillator and two intrinsic thalamic oscillators: thalamocortical neurons and neurons of the TRN[52]. Thalamic neurons are equipped with the biophysical machinery that is necessary to generate a variety of sleep-related rhythms, including slow oscillations, delta oscillations, and sleep spindles. Thalamic UP states have been shown to precede cortical slow oscillations at the level of single cells and the local field potential[43,53], thus suggesting that the thalamic drive might serve as the trigger for cortical UP states[52].

In this context, it is important to note that thalamic neurons can switch between two modes of firing: phasic (bursting) and tonic[54,55,56,57]. Depending on the initial resting membrane potential, these neurons are able to either reliably transmit spike trains or initiate oscillatory burst firing in response to the incoming synaptic drive (or intracellular current injection under experimental conditions). Thalamocortical neurons are able to do that based on the repertoire of voltage-gated ion channels that they express. In particular, the T-type $Ca^{2+}$ channels, which are deinactivated at hyperpolarized membrane potentials (<–65 mV) and initiate a low-threshold $Ca^{2+}$ spike when activated, strongly contribute to this property of thalamocortical neurons[54,55,56,57]. In turn, the resting membrane potential of thalamocortical neurons is heavily influenced by the extracellular neuromodulatory milieu; wake-promoting neuromodulators including acetylcholine and norepinephrine are known to bind surface-expressed G-protein coupled receptors, which lead to an increase in the resting membrane potential of thalamocortical neurons and augmented likelihood of tonic firing[58,59]. Other neuromodulators, such as adenosine, are thought to do the opposite, enhancing burst firing and perhaps the initiation of sleep-related rhythms[60,61].

Slow oscillations influence neuronal activity in different areas of the brain, including the hippocampus. While hippocampal interneurons display a clear bimodal membrane distribution phase-locked to cortical slow oscillations[42], hippocampal pyramidal neurons do not, but their activity is nonetheless profoundly modulated by the slow oscillation[62]. Prominent hippocampal field potential oscillations known as sharp-wave ripple (SWR) complexes, for example, are more likely to occur during cortical UP states[41,63]. Given that these hippocampal events include replayed place cell sequential off-line firing, and that their activity coincides with cortical UP states, it is thought that they may play an important role in the coordination of corticohippocampal activity necessary for memory consolidation[21]. Additionally, UP states appear to coordinate SWR-spindle events, which occur at an increased incidence after learning, further supporting a role of this rhythm in memory consolidation[64,65].

In freely behaving animals, extracellular recordings show frames of high-frequency neuronal firings termed "ON periods" which correlate with the local field potential UP states and "OFF periods" that correlate with the DOWN states[20,21]. As expected, these frames are prevalent in SWS and it appears that the incidence and duration of neuronal OFF periods serve as markers of SWS intensity[20]. Similar to how sleep as a behavioral state is homeostatically regulated (see later sections), both slow oscillations and the corresponding OFF periods of neuronal firing are homeostatically regulated as a function of prior wakefulness. More interestingly, OFF periods of neuronal firing are increased as a function of prior wakefulness, suggesting an important role of this process in brain physiology[20].

An important property of the slow oscillation is that it organizes other sleep rhythms, including delta oscillations and spindles[32]. Spindles, which are 7- to 12-Hz thalamocortical oscillations, are known to be triggered by cortical UP states[66]. It is thought that the cortical drive of the TRN neurons, while they are in a hyperpolarized resting membrane potential, results in triggering membrane potential oscillations at the spindle frequency. This imposes an inhibitory rhythmic synaptic drive onto thalamocortical cells, which ultimately results in entrainment of the cortex to that rhythm[67]. Work by Sara's lab has shown that spindle density rises in the cortex as a function of learning, suggesting that this rhythm might be important for off-line memory processing[68]. Interestingly, while slow oscillations and delta oscillations are low-frequency rhythms that are classically thought to promote synaptic depression, a number of experiments support a role for spindle oscillations in the induction of cortical long-term potentiation[69].

## ASTROCYTES AS NEUROMODULATORS

In 1994, Parpura and colleagues conducted a set of experiments making an intriguing discovery: cultured astrocytes release glutamate, which leads to $Ca^{2+}$ elevation of nearby neurons[70]. Later studies demonstrated that this process can be observed in acute brain slices[71,72,73,74,75,76,77,78,79] and *in vivo*[80]. Astrocytes were later shown to release a number of chemical transmitters, including ATP[81,82,83,84,85], D-serine[86,87,88,89], TNF-alpha[90,91], and ANP[92], in a process that has recently been termed gliotransmission[93]. Based on the early studies of gliotransmission, the concept of the tripartite synapse was proposed[94], highlighting the role of the astrocyte as a third active element in information processing at the synapse[95,96,97,98,99]. Although many aspects of this astrocytes-to-neuron communication are still to be elucidated[89,100] (for reviews, see [101,102,103]), the introduction of molecular genetic tools[83] is shedding light on the neuromodulatory roles of astrocytes on brain function at the level of synapses[83], circuits[104], and behavior[105].

It is important to note that astrocytes can release a number of different chemical transmitters that can have complex, even opposing, effects on neighboring neurons[106]. For example, glutamate and D-serine released from astrocytes can boost NMDA receptor–mediated current, resulting in an excitatory feedback to neurons[107,108]. In contrast, once released from astrocytes, ATP generates one of its metabolites, adenosine, which acts on adenosine A1 receptors to inhibit synaptic transmission[83]. Because of the important role of ATP and its degradation product adenosine, we will focus mainly on this gliotransmitter for the rest of this review.

Although it had been known for quite some time that ATP hydrolysis leads to the accumulation of adenosine, which tonically activates A1 receptors in the hippocampus and the cortex[109,110], its origin was not revealed until the seminal study of Pascual et al. in the hippocampus[83]. The investigators used molecular genetic approaches to perturb gliotransmission. Attenuation of gliotransmission was accomplished by the conditional, astrocyte-specific expression of a cytoplasmic tail of synaptobrevin 2 (lacking the transmembrane domain), which acts as a dominant-negative inhibitor of SNARE-dependent membrane fusion (dnSNARE). Functional studies performed at the hippocampal Schaffer collateral-CA1 synapses revealed that mice expressing dnSNARE in astrocytes showed stronger synaptic transmission compared to wild-type littermates, or transgenic mice in which transgene expression was prevented[83].

Pharmacological approaches aimed at manipulating receptors that respond to known gliotransmitters showed that the observed effect was primarily due to a decrease in extracellular adenosine in slices of the dnSNARE animals. For example, addition of the A1 receptor antagonist 8-cyclopentyl-1,3-dipropylxanthine (DPCPX) to wild-type slices enhanced synaptic transmission, but had no effect when synapses from transgenic mice were studied. Because A1 receptor activation is known to exert tonic suppression of synaptic transmission, the removal of a glial source of adenosine leads to the enhancement of synaptic transmission. A subsequent experiment revealed that dnSNARE overexpression in astrocytes does not perturb the release of adenosine, but rather that of ATP, and that ectonucleotidases hydrolyze ATP to adenosine in the extracellular space[83].

In the hippocampus, high-frequency stimulation of a subset of the Schaffer collateral fibers causes potentiation of the activated synapses (homosynaptic potentiation) and an adenosine-mediated depression of nearby noninnervated synapses (heterosynaptic depression)[111]. Although it has been known that this dynamic process is mediated by adenosine acting through A1 receptors, the cellular source of adenosine had long been undefined. Using transgenic mice expressing dnSNARE in astrocytes, Pascual and colleagues demonstrated that activation of Schaffer collaterals is unable, in these mice, to cause adenosine-mediated heterosynaptic depression[83]. Therefore, in response to synaptic activity, the astrocyte-derived adenosine is augmented to allow a transient depression of neighboring synapses.

In this context, it is interesting to note that Hughes and colleagues demonstrated that infra-slow oscillations, a particular network rhythm at <0.1 Hz recorded in thalamic slices, can be induced by the application of acetylcholine and metabotropic glutamate receptor agonists, and depends on the activation of adenosine A1 receptors[112]. The adenosine modulating the infra-slow oscillations derives from the hydrolysis of ATP and activates $Ba^{2+}$-sensitive $K^+$ channels. Although direct experimental evidence was not provided in that study, the authors suggest that thalamic astrocytes could represent the major source of the ATP/adenosine regulating the infra-slow oscillation[112].

In addition to adenosine, ATP itself can have neuromodulatory effects. For example, in the hypothalamus, Bains' group discovered that ATP released from astrocytes is involved in mediating the action of norepinephrine on AMPAR-mediated synaptic plasticity[113]. Moreover, in a recent study, the same authors observed that activity-dependent ATP release from astrocytes leads to a new form of feed-forward synaptic plasticity on hypothalamic neurons[114]. The authors propose a model in which synaptic release of glutamate activates group I metabotropic glutamate receptors and $Ca^{2+}$ signaling in hypothalamic astrocytes. Following $Ca^{2+}$ elevations, ATP is released by astrocytes, which directly activates P2X receptors in the postsynaptic neuron leading to the net potentiation of glutamatergic synapses. Importantly, the authors argue that the plasticity relies on $Ca^{2+}$ elevations within astrocytic processes, rather than astrocytic cell somata, and demonstrate that it is spatially localized to the neuronal compartment that is in close contact with the activated astrocytes[114].

## SLOW OSCILLATIONS AS A MANIFESATION OF INTEGRATED BRAIN CIRCUIT ACTIVITY

As summarized in the previous section, a large amount of data now supports the view that astrocytes are crucial modulators of synapses, and that their activity can regulate synaptic transmission and plasticity. Nonetheless, whether the astrocytes can modulate neuronal network activity, brain rhythmogenesis *in vivo*, as well as behavior was still an open question. The development of mouse models of impaired gliotransmission offered the opportunity to test these hypotheses.

By using extracellular and patch-clamp recordings in anesthetized animals, we showed that the selective expression of a dominant-negative form of synaptobrevin 2 into astrocytes, to inhibit gliotransmission[83,115], results in decreased slow oscillations in the somatosensory cortex[104] (Fig. 2). The decreased slow oscillation activity is due to the astrocytic modulation of the cortical synapse at least at two different sites. First, a loss of the tonic level of extracellular adenosine–activating A1 receptors and,

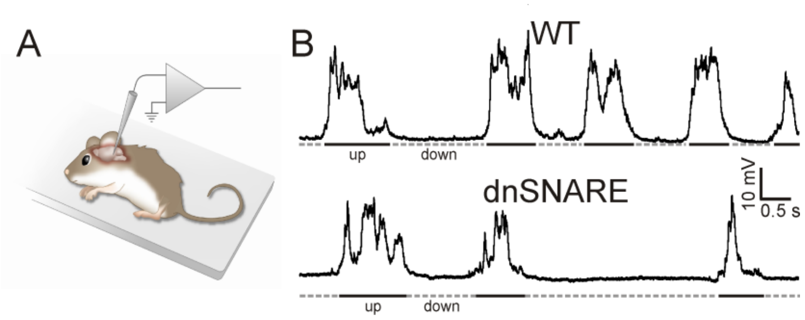

**FIGURE 2.** The non-neuronal cell astrocyte modulates slow oscillations. (A) Schematic representation of the experimental configuration for *in vivo* electrophysiological experiments. (B) Representative patch-clamp recordings from a wild-type animal (top) and a transgenic mouse in which a dominant-negative fragment of synaptobrevin 2 is selectively expressed in astrocytes to inhibit gliotransmission (dnSNARE, bottom). Note the reduced frequency and duration of UP-state transitions in transgenic mice compared to controls. Reproduced with permission from Fellin et al.[104].

second, a decreased function of neuronal NMDA receptors. The hypofunction of cortical NMDA receptors is consistent with a lack of D-serine release from astrocytes and a reduced surface expression of NMDA receptors[104]. Although we could not exclude the possibility that other gliotransmitters may contribute to the observed reduction in slow oscillations, our study was the first to demonstrate the relevance of astrocytic modulation of synapses to the generation of whole network activity *in vivo*. These results point to astrocytes as previously unacknowledged, neuromodulatory components of brain circuits that can deeply influence sleep-related rhythmogenesis.

As mentioned earlier, sleep is a fundamental and evolutionary ancient behavior that is regulated by the circadian oscillator and the sleep homeostat[116,117,118]. The sleep homeostat is the brain process by which sleep intensity is increased as a function of prior wakefulness. The molecular and cellular processes underlying sleep homeostasis have been studied for over 90 years. This investigation started with transfer experiments in which cerebrospinal fluid (CSF) extracts and cerebral venous blood from sleep-deprived animals were injected into control animals to determine whether they can induce sleep. These experiments, aimed at isolating endogenous sleep factors, were largely unsuccessful; however, they were essential in establishing that sleep factors are generated locally within the brain[119,120,121]. More recently, studies have implicated adenosine as an endogenous sleep factor[122,123]. Porkka-Heiskannen and colleagues determined that adenosine levels vary with sleep propensity. That is, during wakefulness, adenosine levels progressively increase, while during sleep they subside. Antagonizing adenosine (both A1 and A2A) receptors promotes wakefulness[124], while injecting adenosine or its agonists into the brain promotes sleep[125,126]. Thus adenosine may not only be a sleep factor, but also a mediator of the homeostatic sleep response.

Given that our studies[104] have shown a role for adenosine of astrocytic origin in the control of cortical slow oscillations, we used the dnSNARE mice to study whether a dysfunctional astrocyte-to-neuron communication could lead to alterations in sleep-related behaviors. By studying the natural sleep behavior of dnSNARE mice with chronic EEG recordings, we observed that these animals show impaired accumulation of slow wave activity (SWA) as a function of prior wakefulness[105]. This deficit is present both under baseline conditions (while animals are undisturbed in their cages) and following sleep deprivation by gentle handling. In accordance with these data, impaired SWA accumulation is also present in animals in which the A1 receptor is genetically deleted from excitatory neurons of the forebrain[127], and these animals have impaired working memory in the face of the chronic sleep loss. In our studies, the SWA phenotype observed in dnSNARE animals is mimicked by pharmacological blockade of A1 receptors in wild-type mice, suggesting that astrocytes represent a source of extracellular adenosine that activates neuronal A1 receptors to control sleep pressure accumulation. Moreover, both dnSNARE animals and their pharmacological phenocopies show resistance of the cognitive effects of short-term sleep deprivation[105]. The opposing roles of purinergic gliotransmission on cognitive function in the short vs. the long term point to intriguing ways in which the brain deals with chronic stressors and how this results in adaptive physiological changes to enhance behavioral performance. Importantly, at the heart of this adaptive response, slow oscillation homeostasis appears to be central.

## CONCLUSIONS

In this review article, we summarized the most recent data aimed at elucidating the cellular determinants of slow oscillations, a fundamental network activity that characterizes NREM sleep, with specific focus on the interaction between neurons and astrocytes. Although slow oscillations have been traditionally viewed as exclusively generated by cortical circuits, a recent hypothesis has proposed this rhythm to originate from the coordinated interaction of three oscillators: two intrinsically bursting thalamic networks (thalamic nucleus and nucleus reticularis thalami) in concert with a synaptic-based cortical oscillator. Based on the available experimental results, we propose an additional level of complexity to this model by considering the glial cell, astrocyte, as a local modulator of both cortical and thalamic circuits (Fig. 3). We argue that only the integrated view of all the neuronal and non-neuronal cellular components in the thalamic and cortical networks will provide a better understanding of the generation and fine tuning of this fundamental brain rhythm.

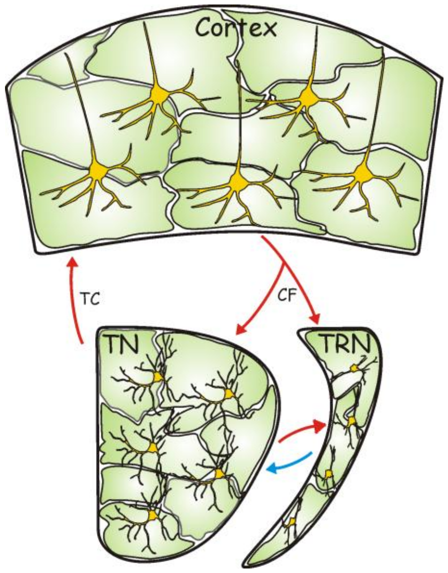

**FIGURE 3.** Slow oscillation generation: an integrated circuit view. Intracortical circuits are fundamental and sufficient to generate slow oscillations. Nonetheless, slow oscillations are likely influenced by the activity of the thalamic nucleus (TN) and the thalamic reticular nucleus (TRN), the targets of corticofugal fibers (CF). The coordinated activity of these two thalamic structures can then modulate cortical activity through the thalamocortical (TC) projections innervating cortical layer IV. We propose that slow oscillations result from the integrated activity of the glial (green cells) and neuronal (yellow cells) networks in the thalamocortical loop. Red and blue arrows indicate excitatory and inhibitory synaptic connections, respectively.

## ACKNOWLEDGMENTS

We thank V. Tucci for critical reading of the manuscript and helpful comments. This work was supported by the MIUR PRIN program, Telethon-Italy (GGP09134), San Paolo grants to FB, grant # R01-NS037585 to PGH, and the San Paolo "Programma in Neuroscienze" to TF.

## References

1. Cirelli, C. and Tononi, G. (2008) Is sleep essential? *PLoS Biol.* **6**, e216.
2. Siegel, J.M. (2008) Do all animals sleep? *Trends Neurosci.* **31**, 208–213.
3. Siegel, J.M. (2009) Sleep viewed as a state of adaptive inactivity. *Nat. Rev. Neurosci*. **10**, 747–753.
4. Meddis, R. (1975) On the function of sleep. *Anim. Behav*. **23**, 676–691.
5. Mignot, E. (2008) Why we sleep: the temporal organization of recovery. *PLoS Biol.* **6**, e106.
6. Diekelmann, S. and Born, J. (2010) The memory function of sleep. *Nat. Rev. Neurosci.* **11**, 114–126.
7. Chee, M.W. and Chuah, L.Y. (2008) Functional neuroimaging insights into how sleep and sleep deprivation affect memory and cognition. *Curr. Opin. Neurol.* **21**, 417–423.
8. Stickgold, R. and Walker, M.P. (2007) Sleep-dependent memory consolidation and reconsolidation. *Sleep Med.* **8**, 331–343.
9. Frank, M.G. and Benington, J.H. (2006) The role of sleep in memory consolidation and brain plasticity: dream or reality? *Neuroscientist* **12**, 477–488.
10. Marshall, L., Helgadottir, H., Molle, M., and Born, J. (2006) Boosting slow oscillations during sleep potentiates memory. *Nature* **444**, 610–613.
11. Rudoy, J.D., Voss, J.L., Westerberg, C.E., and Paller, K.A. (2009) Strengthening individual memories by reactivating them during sleep. *Science* **326**, 1079.
12. Rasch, B., Buchel, C., Gais, S., and Born, J. (2007) Odor cues during slow-wave sleep prompt declarative memory consolidation. *Science* **315**, 1426–1429.
13. Brawn, T.P., Nusbaum, H.C., and Margoliash, D. (2010) Sleep-dependent consolidation of auditory discrimination learning in adult starlings. *J. Neurosci.* **30**, 609–613.
14. Aton, S.J., Seibt, J., Dumoulin, M., Jha, S.K., Steinmetz, N., Coleman, T., Naidoo, N., and Frank, M.G. (2009) Mechanisms of sleep-dependent consolidation of cortical plasticity. *Neuron* **61**, 454–466.
15. Frank, M.G., Issa, N.P., and Stryker, M.P. (2001) Sleep enhances plasticity in the developing visual cortex. *Neuron* **30**, 275–287.
16. Vyazovskiy, V.V., Cirelli, C., Pfister-Genskow, M., Faraguna, U., and Tononi, G. (2008) Molecular and electrophysiological evidence for net synaptic potentiation in wake and depression in sleep. *Nat. Neurosci.* **11**, 200–208.
17. Tononi, G. (2009) Slow wave homeostasis and synaptic plasticity. *J. Clin. Sleep Med.* **5**, S16–S19.
18. Wilson, M.A. and McNaughton, B.L. (1994) Reactivation of hippocampal ensemble memories during sleep. *Science* **265**, 676–679.
19. Suzuki, W.A. (2006) Encoding new episodes and making them stick. *Neuron* **50**, 19–21.
20. Vyazovskiy, V.V., Olcese, U., Lazimy, Y.M., Faraguna, U., Esser, S.K., Williams, J.C., Cirelli, C., and Tononi, G. (2009) Cortical firing and sleep homeostasis. *Neuron* **63**, 865–878.
21. Ji, D.Y. and Wilson, M.A. (2007) Coordinated memory replay in the visual cortex and hippocampus during sleep. *Nat. Neurosci.* **10**, 100–107.
22. Louie, K. and Wilson, M.A. (2001) Temporally structured replay of awake hippocampal ensemble activity during rapid eye movement sleep. *Neuron* **29**, 145–156.
23. Nadasdy, Z., Hirase, H., Czurko, A., Csicsvari, J., and Buzsaki, G. (1999) Replay and time compression of recurring spike sequences in the hippocampus. *J. Neurosci.* **19**, 9497–9507.
24. Maquet, P. et al. (2000) Experience-dependent changes in cerebral activation during human REM sleep. *Nat. Neurosci.* **3**, 831–836.
25. Popa, D., Duvarci, S., Popescu, A.T., Lena, C., and Pare, D. (2010) Coherent amygdalocortical theta promotes fear memory consolidation during paradoxical sleep. *Proc. Natl. Acad. Sci. U. S. A.* **107,** 6516–6519.
26. Lee, A.K. and Wilson, M.A. (2002) Memory of sequential experience in the hippocampus during slow wave sleep. *Neuron* **36**, 1183–1194.
27. Molle, M., Marshall, L., Gais, S., and Born, J. (2004) Learning increases human electroencephalographic coherence during subsequent slow sleep oscillations. *Proc. Natl. Acad. Sci. U. S. A.* **101**, 13963–13968.
28. Huber, R., Ghilardi, M.F., Massimini, M., and Tononi, G. (2004) Local sleep and learning. *Nature* **430**, 78–81.
29. Steriade, M., Contreras, D., Dossi, R.C., and Nunez, A. (1993) The slow (less-than-1 Hz) oscillation in reticular thalamic and thalamocortical neurons - scenario of sleep rhythm generation in interacting thalamic and neocortical networks. *J. Neurosci.* **13**, 3284–3299.

30. Steriade, M., Nunez, A., and Amzica, F. (1993) Intracellular analysis of relations between the slow (less-than-1 Hz) neocortical oscillation and other sleep rhythms of the electroencephalogram. *J. Neurosci.* **13**, 3266–3283.
31. Steriade, M., Nunez, A., and Amzica, F. (1993) A novel slow (less-than-1 Hz) oscillation of neocortical neurons in-vivo - depolarizing and hyperpolarizing components. *J. Neurosci.* **13**, 3252–3265.
32. Steriade, M. (2006) Grouping of brain rhythms in corticothalamic systems. *Neuroscience* **137**, 1087–1106.
33. Sirota, A. and Buzsaki, G. (2005) Interaction between neocortical and hippocampal networks via slow oscillations. *Thalamus. Relat. Syst.* **3**, 245–259.
34. Cash, S.S. et al. (2009) The human K-complex represents an isolated cortical down-state. *Science* **324**, 1084–1087.
35. Marshall, L. and Born, J. (2007) The contribution of sleep to hippocampus-dependent memory consolidation. *Trends Cogn. Sci.* **11**, 442–450.
36. Massimini, M., Huber, R., Ferrarelli, F., Hill, S., and Tononi, G. (2004) The sleep slow oscillation as a traveling wave. *J. Neurosci.* **24**, 6862–6870.
37. Petersen, C.C., Hahn, T.T., Mehta, M., Grinvald, A., and Sakmann, B. (2003) Interaction of sensory responses with spontaneous depolarization in layer 2/3 barrel cortex. *Proc. Natl. Acad. Sci. U. S. A.* **100**, 13638–13643.
38. Luczak, A., Bartho, P., Marguet, S.L., Buzsaki, G., and Harris, K.D. (2007) Sequential structure of neocortical spontaneous activity in vivo. *Proc. Natl. Acad. Sci. U. S. A.* **104**, 347–352.
39. Mohajerani, M.H., McVea, D.A., Fingas, M., and Murphy, T.H. (2010) Mirrored bilateral slow-wave cortical activity within local circuits revealed by fast bihemispheric voltage-sensitive dye imaging in anesthetized and awake mice. *J. Neurosci.* **30**, 3745–3751.
40. Isomura, Y., Sirota, A., Ozen, S., Montgomery, S., Mizuseki, K., Henze, D.A., and Buzsaki, G. (2006) Integration and segregation of activity in entorhinal-hippocampal subregions by neocortical slow oscillations. *Neuron* **52**, 871–882.
41. Sirota, A., Csicsvari, J., Buhl, D., and Buzsaki, G. (2003) Communication between neocortex and hippocampus during sleep in rodents. *Proc. Natl. Acad. Sci. U. S. A.* **100**, 2065–2069.
42. Hahn, T.T., Sakmann, B., and Mehta, M.R. (2006) Phase-locking of hippocampal interneurons' membrane potential to neocortical up-down states. *Nat. Neurosci.* **9**, 1359–1361.
43. Contreras, D. and Steriade, M. (1995) Cellular basis of EEG slow rhythms: a study of dynamic corticothalamic relationships. *J. Neurosci.* **15**, 604–622.
44. Contreras, D. and Steriade, M. (1997) Synchronization of low-frequency rhythms in corticothalamic networks. *Neuroscience* **76**, 11–24.
45. Steriade, M. (1997) Synchronized activities of coupled oscillators in the cerebral cortex and thalamus at different levels of vigilance. *Cereb. Cortex* **7**, 583–604.
46. Hughes, S.W., Cope, D.W., Blethyn, K.L., and Crunelli, V. (2002) Cellular mechanisms of the slow (< 1 Hz) oscillation in thalamocortical neurons in vitro. *Neuron* **33**, 947–958.
47. Sanchez-Vives, M.V. and McCormick, D.A. (2000) Cellular and network mechanisms of rhythmic recurrent activity in neocortex. *Nat. Neurosci.* **3**, 1027–1034.
48. Sakata, S. and Harris, K.D. (2009) Laminar structure of spontaneous and sensory-evoked population activity in auditory cortex. *Neuron* **64**, 404–418.
49. MacLean, J.N., Watson, B.O., Aaron, G.B., and Yuste, R. (2005) Internal dynamics determine the cortical response to thalamic stimulation. *Neuron* **48**, 811–823.
50. Shu, Y.S., Hasenstaub, A., and McCormick, D.A. (2003) Turning on and off recurrent balanced cortical activity. *Nature* **423**, 288–293.
51. Timofeev, I., Grenier, F., Bazhenov, M., Sejnowski, T.J., and Steriade, M. (2000) Origin of slow cortical oscillations in deafferented cortical slabs. *Cereb. Cortex* **10**, 1185–1199.
52. Crunelli, V. and Hughes, S.W. (2010) The slow (<1 Hz) rhythm of non-REM sleep: a dialogue between three cardinal oscillators. *Nat. Neurosci.* **13**, 9–17.
53. Rigas, P. and Castro-Alamancos, M.A. (2007) Thalamocortical Up states: differential effects of intrinsic and extrinsic cortical inputs on persistent activity. *J. Neurosci.* **27**, 4261–4272.
54. Eccles, J.C. (1965) Inhibition in thalamic and cortical neurones and its role in phasing neuronal discharges. *Epilepsia* **6**, 89–115.
55. Deschenes, M., Paradis, M., Roy, J.P., and Steriade, M. (1984) Electrophysiology of neurons of lateral thalamic nuclei in cat: resting properties and burst discharges. *J. Neurophysiol.* **51**, 1196–1219.
56. Jahnsen, H. and Llinas, R. (1984) Electrophysiological properties of guinea-pig thalamic neurones: an in vitro study. *J. Physiol.* **349**, 205–226.
57. Contreras, D. (2006) The role of T-channels in the generation of thalamocortical rhythms. *CNS Neurol. Disord. Drug Targets* **5**, 571–585.
58. McCormick, D.A. (1992) Neurotransmitter actions in the thalamus and cerebral cortex and their role in neuromodulation of thalamocortical activity. *Prog. Neurobiol.* **39**, 337–388.
59. Broicher, T., Wettschureck, N., Munsch, T., Coulon, P., Meuth, S.G., Kanyshkova, T., Seidenbecher, T., Offermanns, S., Pape, H.C., and Budde, T. (2008) Muscarinic ACh receptor-mediated control of thalamic activity via G(q)/G (11)-family G-proteins. *Pflugers Arch.* **456**, 1049–1060.
60. Wallenstein, G.V. (1996) Adenosinic modulation of 7-14 Hz spindle rhythms in interconnected thalamic relay and nucleus reticularis neurons. *Neuroscience* **73**, 93–98.

61. Bjorness, T.E. and Greene, R.W. (2009) Adenosine and sleep. *Curr. Neuropharmacol.* **7**, 238–245.
62. Hahn, T.T., Sakmann, B., and Mehta, M.R. (2007) Differential responses of hippocampal subfields to cortical up-down states. *Proc. Natl. Acad. Sci. U. S. A.* **104**, 5169–5174.
63. Siapas, A.G. and Wilson, M.A. (1998) Coordinated interactions between hippocampal ripples and cortical spindles during slow-wave sleep. *Neuron* **21**, 1123–1128.
64. Eschenko, O., Molle, M., Born, J., and Sara, S.J. (2006) Elevated sleep spindle density after learning or after retrieval in rats. *J. Neurosci.* **26**, 12914–12920.
65. Molle, M., Eschenko, O., Gais, S., Sara, S.J., and Born, J. (2009) The influence of learning on sleep slow oscillations and associated spindles and ripples in humans and rats. *Eur. J. Neurosci.* **29**, 1071–1081.
66. Contreras, D. and Steriade, M. (1996) Spindle oscillation in cats: the role of corticothalamic feedback in a thalamically generated rhythm. *J. Physiol* **490(Pt 1)**, 159–179.
67. Hartwich, K., Pollak, T., and Klausberger, T. (2009) Distinct firing patterns of identified basket and dendrite-targeting interneurons in the prefrontal cortex during hippocampal theta and local spindle oscillations. *J. Neurosci.* **29**, 9563–9574.
68. Eschenko, O., Molle, M., Born, J., and Sara, S.J. (2006) Elevated sleep spindle density after learning or after retrieval in rats. *J. Neurosci.* **26**, 12914–12920.
69. Rosanova, M. and Ulrich, D. (2005) Pattern-specific associative long-term potentiation induced by a sleep spindle-related spike train. *J. Neurosci.* **25**, 9398–9405.
70. Parpura, V., Basarsky, T.A., Liu, F., Jeftinija, K., Jeftinija, S., and Haydon, P.G. (1994) Glutamate-mediated astrocyte-neuron signalling. *Nature* **369**, 744–747.
71. Bezzi, P., Carmignoto, G., Pasti, L., Vesce, S., Rossi, D., Rizzini, B.L., Pozzan, T., and Volterra, A. (1998) Prostaglandins stimulate calcium-dependent glutamate release in astrocytes. *Nature* **391**, 281–285.
72. Pasti, L., Zonta, M., Pozzan, T., Vicini, S., and Carmignoto, G. (2001) Cytosolic calcium oscillations in astrocytes may regulate exocytotic release of glutamate. *J. Neurosci.* **21**, 477–484.
73. Fellin, T., Pascual, O., Gobbo, S., Pozzan, T., Haydon, P.G., and Carmignoto, G. (2004) Neuronal synchrony mediated by astrocytic glutamate through activation of extrasynaptic NMDA receptors. *Neuron* **43**, 729–743.
74. Angulo, M.C., Kozlov, A.S., Charpak, S., and Audinat, E. (2004) Glutamate released from glial cells synchronizes neuronal activity in the hippocampus. *J. Neurosci*. **24**, 6920–6927.
75. Perea, G. and Araque, A. (2005) Properties of synaptically evoked astrocyte calcium signal reveal synaptic information processing by astrocytes. *J. Neurosci.* **25**, 2192–2203.
76. Tian, G.F., Azmi, H., Takano, T., Xu, Q., Peng, W., Lin, J., Oberheim, N., Lou, N., Wang, X., Zielke, H.R., Kang, J., and Nedergaard, M. (2005) An astrocytic basis of epilepsy. *Nat. Med.* **11**, 973–981.
77. Perea, G. and Araque, A. (2007) Astrocytes potentiate transmitter release at single hippocampal synapses. *Science* **317**, 1083–1086.
78. Jourdain, P., Bergersen, L.H., Bhaukaurally, K., Bezzi, P., Santello, M., Domercq, M., Matute, C., Tonello, F., Gundersen, V., and Volterra, A. (2007) Glutamate exocytosis from astrocytes controls synaptic strength. *Nat. Neurosci.* **10**, 331–339.
79. Navarrete, M. and Araque, A. (2008) Endocannabinoids mediate neuron-astrocyte communication. *Neuron* **57**, 883–893.
80. Ding, S., Fellin, T., Zhu, Y., Lee, S.Y., Auberson, Y.P., Meaney, D.F., Coulter, D.A., Carmignoto, G., and Haydon, P.G. (2007) Enhanced astrocytic Ca2+ signals contribute to neuronal excitotoxicity after status epilepticus. *J. Neurosci.* **27**, 10674–10684.
81. Cotrina, M.L., Lin, J.H., Lopez-Garcia, J.C., Naus, C.C., and Nedergaard, M. (2000) ATP-mediated glia signaling. *J. Neurosci.* **20**, 2835–2844.
82. Zhang, J.M., Wang, H.K., Ye, C.Q., Ge, W., Chen, Y., Jiang, Z.L., Wu, C.P., Poo, M.M., and Duan, S. (2003) ATP released by astrocytes mediates glutamatergic activity-dependent heterosynaptic suppression. *Neuron* **40**, 971–982.
83. Pascual, O., Casper, K.B., Kubera, C., Zhang, J., Revilla-Sanchez, R., Sul, J.Y., Takano, H., Moss, S.J., McCarthy, K., and Haydon, P.G. (2005) Astrocytic purinergic signaling coordinates synaptic networks. *Science* **310**, 113–116.
84. Serrano, A., Haddjeri, N., Lacaille, J.C., and Robitaille, R. (2006) GABAergic network activation of glial cells underlies hippocampal heterosynaptic depression. *J. Neurosci.* **26**, 5370–5382.
85. Andersson, M., Blomstrand, F., and Hanse, E. (2007) Astrocytes play a critical role in transient heterosynaptic depression in the rat hippocampal CA1 region. *J. Physiol.* **585**, 843–852.
86. Yang, Y., Ge, W., Chen, Y., Zhang, Z., Shen, W., Wu, C., Poo, M., and Duan, S. (2003) Contribution of astrocytes to hippocampal long-term potentiation through release of D-serine. *Proc. Natl. Acad. Sci. U. S. A.* **100**, 15194–15199.
87. Mothet, J.P., Pollegioni, L., Ouanounou, G., Martineau, M., Fossier, P., and Baux, G. (2005) Glutamate receptor activation triggers a calcium-dependent and SNARE protein-dependent release of the gliotransmitter D-serine. *Proc. Natl. Acad. Sci. U. S. A.* **102**, 5606–5611.
88. Panatier, A., Theodosis, D.T., Mothet, J.P., Touquet, B., Pollegioni, L., Poulain, D.A., and Oliet, S.H.R. (2006) Glia-derived D-serine controls NMDA receptor activity and synaptic memory. *Cell* **125**, 775–784.
89. Henneberger, C., Papouin, T., Oliet, S.H., and Rusakov, D.A. (2010) Long-term potentiation depends on release of D-serine from astrocytes. *Nature* **463**, 232–236.

90. Beattie, E.C., Stellwagen, D., Morishita, W., Bresnahan, J.C., Ha, B.K., Von Zastrow, M., Beattie, M.S., and Malenka, R.C. (2002) Control of synaptic strength by glial TNF alpha. *Science* **295**, 2282–2285.
91. Stellwagen, D. and Malenka, R.C. (2006) Synaptic scaling mediated by glial TNF-alpha. *Nature* **440**, 1054–1059.
92. Krzan, M., Stenovec, M., Kreft, M., Pangrsic, T., Grilc, S., Haydon, P.G., and Zorec, R. (2003) Calcium-dependent exocytosis of atrial natriuretic peptide from astrocytes. *J. Neurosci.* **23**, 1580–1583.
93. Zhang, Q. and Haydon, P.G. (2005) Roles for gliotransmission in the nervous system. *J. Neural Transm.* **112**, 121–125.
94. Araque, A., Parpura, V., Sanzgiri, R.P., and Haydon, P.G. (1999) Tripartite synapses: glia, the unacknowledged partner. *Trends Neurosci.* **22**, 208–215.
95. Halassa, M.M., Fellin, T., and Haydon, P.G. (2007) The tripartite synapse: roles for gliotransmission in health and disease. *Trends Mol. Med.* **13**, 54–63.
96. Halassa, M.M., Fellin, T., and Haydon, P.G. (2009) Tripartite synapses: roles for astrocytic purines in the control of synaptic physiology and behavior. *Neuropharmacology* **57**, 343–346.
97. Fellin, T. (2009) Communication between neurons and astrocytes: relevance to the modulation of synaptic and network activity. *J. Neurochem*. **108**, 533–544.
98. Perea, G., Navarrete, M., and Araque, A. (2009) Tripartite synapses: astrocytes process and control synaptic information. *Trends Neurosci*. **32**, 421–431.
99. Halassa, M.M. and Haydon, P.G. (2010) Integrated brain circuits: astrocytic networks modulate neuronal activity and behavior. *Annu. Rev. Physiol*. **72**, 335–355.
100. Agulhon, C., Fiacco, T.A., and McCarthy, K.D. (2010) Hippocampal short- and long-term plasticity are not modulated by astrocyte Ca2+ signaling. *Science* **327**, 1250–1254.
101. Haydon, P.G. and Carmignoto, G. (2006) Astrocyte control of synaptic transmission and neurovascular coupling. *Physiol. Rev.* **86**, 1009–1031.
102. Hamilton, N.B. and Attwell, D. (2010) Do astrocytes really exocytose neurotransmitters? *Nat. Rev. Neurosci.* **11**, 227–238.
103. Agulhon, C., Petravicz, J., McMullen, A.B., Sweger, E.J., Minton, S.K., Taves, S.R., Casper, K.B., Fiacco, T.A., and McCarthy, K.D. (2008) What is the role of astrocyte calcium in neurophysiology? *Neuron* **59**, 932–946.
104. Fellin, T., Halassa, M.M., Terunuma, M., Succol, F., Takano, H., Frank, M., Moss, S.J., and Haydon, P.G. (2009) Endogenous nonneuronal modulators of synaptic transmission control cortical slow oscillations in vivo. *Proc. Natl. Acad. Sci. U. S. A.* **106**, 15037–15042.
105. Halassa, M.M., Florian, C., Fellin, T., Munoz, J.R., Lee, S.Y., Abel, T., Haydon, P.G., and Frank, M.G. (2009) Astrocytic modulation of sleep homeostasis and cognitive consequences of sleep loss. *Neuron* **61**, 213–219.
106. Fellin, T., Pascual, O., and Haydon, P.G. (2006) Astrocytes coordinate synaptic networks: balanced excitation and inhibition. *Physiology* **21**, 208–215.
107. Volterra, A. and Meldolesi, J. (2005) Astrocytes, from brain glue to communication elements: the revolution continues. *Nat. Rev. Neurosci.* **6**, 626–640.
108. Bains, J.S. and Oliet, S.H. (2007) Glia: they make your memories stick! *Trends Neurosci.* **30**, 417–424.
109. Cunha, R.A., Sebastiao, A.M., and Ribeiro, J.A. (1998) Inhibition by ATP of hippocampal synaptic transmission requires localized extracellular catabolism by ecto-nucleotidases into adenosine and channeling to adenosine A1 receptors. *J. Neurosci.* **18**, 1987–1995.
110. Dunwiddie, T.V., Diao, L., and Proctor, W.R. (1997) Adenine nucleotides undergo rapid, quantitative conversion to adenosine in the extracellular space in rat hippocampus. *J. Neurosci.* **17**, 7673–7682.
111. Manzoni, O.J., Manabe, T., and Nicoll, R.A. (1994) Release of adenosine by activation of NMDA receptors in the hippocampus. *Science* **265**, 2098–2101.
112. Lorincz, M.L., Geall, F., Bao, Y., Crunelli, V., and Hughes, S.W. (2009) ATP-dependent infra-slow (<0.1 Hz) oscillations in thalamic networks. *PLoS One* **4**, e4447.
113. Gordon, G.R., Baimoukhametova, D.V., Hewitt, S.A., Rajapaksha, W.R., Fisher, T.E., and Bains, J.S. (2005) Norepinephrine triggers release of glial ATP to increase postsynaptic efficacy. *Nat. Neurosci.* **8**, 1078–1086.
114. Gordon, G.R., Iremonger, K.J., Kantevari, S., Ellis-Davies, G.C., MacVicar, B.A., and Bains, J.S. (2009) Astrocyte-mediated distributed plasticity at hypothalamic glutamate synapses. *Neuron* **64**, 391–403.
115. Zhang, Q., Pangrsic, T., Kreft, M., Krzan, M., Li, N., Sul, J.Y., Halassa, M., Van Bockstaele, E., Zorec, R., and Haydon, P.G. (2004) Fusion-related release of glutamate from astrocytes. *J. Biol. Chem*. **279**, 12724–12733.
116. Borbely, A.A. (1998) Processes underlying sleep regulation. *Horm. Res*. **49**, 114–117.
117. Borbely, A.A. and Achermann, P. (1999) Sleep homeostasis and models of sleep regulation. *J. Biol. Rhythms* **14**, 557–568.
118. Borbely, A.A. (2001) From slow waves to sleep homeostasis: new perspectives. *Arch. Ital. Biol.* **139**, 53–61.
119. Krueger, J.M. and Obal, F., Jr. (2003) Sleep function. *Front. Biosci.* **8**, d511–d519.
120. Liao, F., Taishi, P., Churchill, L., Urza, M.J., and Krueger, J.M. (2010) Localized suppression of cortical growth hormone-releasing hormone receptors state-specifically attenuates electroencephalographic delta waves. *J. Neurosci.* **30**, 4151–4159.
121. Krueger, J.M., Rector, D.M., Roy, S., Van Dongen, H.P., Belenky, G., and Panksepp, J. (2008) Sleep as a fundamental property of neuronal assemblies. *Nat. Rev. Neurosci.* **9**, 910–919.

122. Porkka-Heiskanen, T., Alanko, L., Kalinchuk, A., and Stenberg, D. (2002) Adenosine and sleep. *Sleep Med. Rev*. **6**, 321–332.
123. Porkka-Heiskanen, T., Kalinchuk, A., Alanko, L., Urrila, A., and Stenberg, D. (2003) Adenosine, energy metabolism, and sleep. *TheScientificWorldJOURNAL* **3**, 790–798.
124. Porkka-Heiskanen, T., Strecker, R.E., Thakkar, M., Bjorkum, A.A., Greene, R.W., and McCarley, R.W. (1997) Adenosine: a mediator of the sleep-inducing effects of prolonged wakefulness. *Science* **276**, 1265–1268.
125. Basheer, R., Porkka-Heiskanen, T., Stenberg, D., and McCarley, R.W. (1999) Adenosine and behavioral state control: adenosine increases c-Fos protein and AP1 binding in basal forebrain of rats. *Brain Res. Mol. Brain Res*. **73**, 1–10.
126. Portas, C.M., Thakkar, M., Rainnie, D.G., Greene, R.W., and McCarley, R.W. (1997) Role of adenosine in behavioral state modulation: a microdialysis study in the freely moving cat. *Neuroscience* **79**, 225–235.
127. Bjorness, T.E., Kelly, C.L., Gao, T., Poffenberger, V., and Greene, R.W. (2009) Control and function of the homeostatic sleep response by adenosine A1 receptors. *J. Neurosci.* **29**, 1267–1276.
